# Does Levosimendan hasten veno-arterial ECMO weaning? A propensity score matching analysis

**DOI:** 10.1186/s13613-025-01457-9

**Published:** 2025-04-03

**Authors:** Nicolas Paulo, Antoine Kimmoun, David Hajage, Pierre Hubert, David Levy, Marc Pineton de Chambrun, Juliette Chommeloux, Ouriel Saura, Grégoire Del Marmol, Quentin Moyon, Guillaume Hékimian, Melchior Gautier, Charles Edouard Luyt, Guillaume Lebreton, Bruno Levy, Alain Combes, Matthieu Schmidt

**Affiliations:** 1 AP-HP, Service de Médecine Intensive-Réanimation, Institut de Cardiologie, Hôpital Pitié-Salpêtrière, 47 Boulevard de L’Hôpital, 75013 Paris, France; 2 Institut Lorrain du Cœur Et Des Vaisseaux, Service de Médecine Intensive-Réanimation, FCRIN-INICRCT, Université de Lorraine, CHRU de Nancy, U1116 Nancy, France; 3 Département Biostatistique Santé Publique Et Information Médicale, Centre de Pharmacoépidémiologie (Cephepi), Sorbonne Université, AP-HP, Hôpital Pitié-Salpêtrière, Unité de Recherche Clinique PSL-CFX, CIC-1421 Paris, France; 4 Institute of Cardiometabolism and Nutrition, Sorbonne Université, UMRS_1166-ICAN75013 Paris, France; 5 AP-HP, Cardiac Surgery Department, Institut de Cardiologie, Hôpital Pitié-Salpêtrière, 75013 Paris, France; 6 Sorbonne Université, GRC 30 RESPIRE, Paris, France

**Keywords:** Extracorporeal membrane oxygenation, Cardiogenic shock—mechanical circulatory support, ECMO weaning, Levosimendan

## Abstract

**Background:**

Preliminary evidence from small, single-center studies suggests levosimendan may improve the likelihood of successful venoarterial extracorporeal membrane oxygenation (VA-ECMO) weaning in patients with cardiogenic shock. However, the literature is limited and presents conflicting results. We aimed to assess the benefits of levosimendan on VA-ECMO for time to successful ECMO weaning, using a pragmatic and rigorous definition of successful VA-ECMO weaning in patients with potential for cardiac function recovery.

**Methods:**

A retrospective bicentric study over 6 years was conducted, including patients who received levosimendan during their ECMO course. Patients with post-cardiotomy cardiogenic shock or end-stage chronic heart failure were excluded. Patients receiving levosimendan while on VA-ECMO were matched to those not receiving levosimendan during the same period, based on pre-specified variables and time from ECMO initiation. The primary endpoint was successful VA-ECMO weaning, defined as survival without death, heart transplantation, or LVAD within 30 days after VA-ECMO withdrawal.

**Results:**

Over the study period, 320 patients treated with VA-ECMO for refractory cardiogenic shock were included, of whom 68 received levosimendan during their ECMO course. Propensity score matching yielded 47 unique pairs of patients with comparable characteristics. After matching, successful ECMO weaning was achieved in 16 out of 47 patients (34%) in the no-levosimendan group and 21 out of 47 patients (45%) in the levosimendan group (sHR, 1.45 [95% CI, 0.77–2.70]; P = 0.25). Similarly, there were no significant differences between the groups in terms of bridge-to-heart transplant, LVAD, or death. Left ventricular ejection fraction and aortic velocity time integral improved significantly after levosimendan in all patients, regardless of their VA-ECMO weaning status.

**Conclusion:**

In patients with non-postoperative cardiogenic shock supported by peripheral VA-ECMO, levosimendan was not associated with increased rates of successful VA-ECMO weaning or improved 30-day and 6-month bridge-free survival. Results from double-blinded randomized controlled trials are urgently needed to clarify the effectiveness and optimal timing of levosimendan in this specific population.

**Supplementary Information:**

The online version contains supplementary material available at 10.1186/s13613-025-01457-9.

## Background

Peripheral venoarterial extracorporeal membrane oxygenation (VA-ECMO) is commonly employed as the primary therapy for refractory cardiogenic shock [1]. Although VA-ECMO offers rapid and effective cardiocirculatory support at the bedside, patient outcomes remain poor, with in-hospital mortality rates ranging from 49 to 50% as reported in recent randomized controlled trials [2, 3]. The process of VA-ECMO weaning presents a significant challenge for clinicians. Levosimendan may improve endothelial function and cardiac output in VA-ECMO patients with severely reduced LVEF, potentially aiding the weaning process. Its distinct inotropic mechanism compared to dobutamine and ECMO’s ability to enhance hemodynamic tolerance may reduce ECMO duration and related complications [4]. Preliminary evidence from small, single-center studies suggests that levosimendan may improve the likelihood of successful VA-ECMO weaning in patients with VA-ECMO-supported cardiogenic shock [5–7], potentially avoiding the need for heart transplantation, total artificial heart implantation, left ventricular assist device (LVAD) insertion, or transition to comfort care. However, the literature is limited and presents conflicting results [5, 8], although recent meta-analyses have indicated a significant association between levosimendan during VA-ECMO and successful weaning and improved survival in patients with cardiogenic shock [9–12]. The heterogeneity in the etiologies of cardiogenic shock (e.g., postcardiotomy, acute myocardial infarction, end-stage chronic cardiomyopathy…), inconsistent definitions of "successful VA-ECMO weaning," and variations in statistical matching approaches may account for these discrepancies. Additionally, there remains a lack of evidence from randomized trials specifically evaluating the use of levosimendan in VA-ECMO weaning.

The physiological rationale for using levosimendan in the context of VA-ECMO weaning appears compelling. This drug enhances cardiac output and stroke volume while reducing peripheral vascular resistance without increasing myocardial oxygen demand. Despite these benefits, recent large, randomized trials have demonstrated that this calcium-sensitizing inotrope and ATP-sensitive potassium-channel opener is ineffective in preventing and treating low cardiac output syndrome following cardiac surgery [13–15]. Given that the effects of levosimendan manifest several hours after infusion initiation, and considering promising studies in this context [5–7], levosimendan is often administered to patients with severe cardiogenic shock on VA-ECMO, particularly when there is a delay in cardiac function recovery [16]. We hypothesize that levosimendan may facilitate successful VA-ECMO weaning in medical patients where VA-ECMO serves as a bridge to recovery. Therefore, our study aims to evaluate the impact of levosimendan on the time to successful VA-ECMO weaning in a large bicentric cohort, utilizing a pragmatic and rigorous definition of successful VA-ECMO weaning in patients with the potential for cardiac function recovery.

## Methods

### Setting

We retrospectively reviewed the ECMO databases from two French university ECMO-referral centers—namely, the Intensive Care Units (ICUs) at La Pitié Salpêtrière Hospital in Paris and Brabois Hospital in Nancy. Our goal was to identify all patients supported with peripheral VA-ECMO for a non-surgical refractory cardiogenic shock and who received levosimendan between October 1, 2014, and June 31, 2018. Patients treated with VA-ECMO after June 2018 were excluded due to our centers' routine use of levosimendan and participation in the double-blinded LEVOECMO randomized controlled trial (NCT04728932). Patients who were on central VA-ECMO and had a post-cardiotomy cardiogenic shock, or an end-stage chronic heart failure (i.e., those with a low likelihood of VA-ECMO weaning) were also excluded. Additionally, patients on VA-ECMO who did not receive levosimendan during the same period were selected from the ENCOURAGE [17], intra-aortic balloon pump on VA-ECMO [18], and acute decompensated heart failure on VA-ECMO [19] cohorts. Following our hospital’s institutional review board ethical standards, informed consent for the analysis of demographic, physiological, and hospital outcome data was not obtained, as this observational study did not alter existing diagnostic or therapeutic strategies. The National Commission for Informatics and Liberties approved this study (no. 1950673).

### VA-ECMO Management

Cardiogenic shock was defined as a sustained systolic blood pressure of < 90 mmHg or the need for vasoactive drugs to maintain systolic blood pressure ≥ 90 mmHg, accompanied by evidence of tissue hypoperfusion, following the latest European Society of Cardiology guidelines [20, 21]. Refractory cardiogenic shock was diagnosed when acute cardiovascular failure persisted despite high-dose catecholamine therapy (e.g., epinephrine ≥ 1 µg/kg/min, dobutamine ≥ 15 µg/kg/min, with or without norepinephrine ≥ 1 µg/kg/min). The management of patients receiving VA-ECMO in our ICUs has been previously detailed [22]. All VA-ECMO devices were inserted either surgically or percutaneously using femoral–femoral cannulation with 23F to 29F venous and 15F to 18F arterial cannula. A 7F catheter was routinely placed in the femoral artery to prevent limb ischemia. The pump speed was adjusted to achieve a blood flow of 3.5–4.5 L/min. Intravenous unfractionated heparin was administered to maintain the activated partial thromboplastin time at 1.5–2 times the normal value. Patients were assessed daily for potential VA-ECMO weaning using clinical and echocardiographic criteria as previously described [23, 24]. After multiple failed VA-ECMO weaning attempts, options such as heart transplantation or long-term mechanical support were considered. If the patient was not eligible for these options, withdrawal of care and transition to comfort care were provided.

### Levosimendan management

In our departments, levosimendan was typically initiated in hemodynamically stabilized patients when there was still potential for cardiac recovery. If a decision to proceed with heart transplantation or the use of an LVAD had been made, either before or after VA-ECMO initiation, levosimendan was not administered. Levosimendan was infused continuously over 24 h without an initial bolus. The infusion was started at a rate of 0.1 µg/kg/min (using 12.5 mg diluted in 50 ml of 0.9% NaCl) and was increased to 0.2 µg/kg/min after 2 h, provided no rate-limiting side effects occurred. The decision to continue dobutamine during levosimendan infusion and the timing of levosimendan initiation were left to the discretion of the treating physician.

### Data Collection

All data related to VA-ECMO implantation and follow-up were retrieved from our ECMO databases and patients' electronic medical records. An inotropic score was calculated using the formula: dobutamine dose (µg/kg/min) + [epinephrine dose (µg/kg/min) + norepinephrine dose (µg/kg/min)] × 100, to quantify inotrope use at the time of VA-ECMO implantation and levosimendan initiation [22]. Left ventricular ejection fraction (LVEF) and aortic velocity time integral (AoVTI) were recorded before cannulation, 24 h before levosimendan administration, 48 h after levosimendan, and at the time of VA-ECMO removal.

### Definitions and Study Endpoints

The primary endpoint was successful VA-ECMO weaning, defined as survival without death, heart transplantation, or LVAD within 30 days after VA-ECMO withdrawal. Secondary endpoints included 30-day and 6-month survival without any bridge, overall 30-day and 6-month survival, heart transplantation, LVAD, and total artificial heart. Additional data collected included the number of ECMO days, lengths of stay in the ICU and hospital, and levosimendan-related complications (e.g., severe hypotension, supraventricular rhythm disorders, and premature discontinuation). Severe hypotension was defined as requiring fluid infusion or an increase in vasopressor doses by more than 50%.

### Statistical Analysis

Descriptive statistics were presented as frequencies (percentages) for categorical variables and medians with interquartile ranges for continuous variables. Proportions were compared using the chi-square and Fisher's exact tests, while continuous variables were compared using the Student's t-test or the Wilcoxon rank-sum.

Matched cohort analyses were conducted using propensity score matching. Variables selected for matching were those anticipated a priori to be associated with ICU mortality in patients with refractory cardiogenic shock on VA-ECMO. The chosen covariates included age, SOFA score at ICU admission, cardiogenic shock etiology, pre-ECMO lactate level, and VA-ECMO use during cardiopulmonary resuscitation (E-CPR). Patients were matched on the specified variables and using a 1:1 propensity score matching procedure without replacement, with a caliper width of 0.1, resulting in minimal differences between matched variables as recommended [25]. Additionally, to avoid immortal time bias, patients receiving levosimendan while on VA-ECMO were matched only to those not receiving levosimendan at a similar point in their illness by aligning the number of days on VA-ECMO before levosimendan administration with an equivalent or greater number of VA-ECMO days in the control group. Robust standard errors (with pair membership as the clustering variable) were used for all outcome comparisons of matched patients.

The primary endpoint was the time to successful weaning from VA-ECMO, accounting for the competing risks of death, bridge to heart transplantation, LVAD, or total artificial heart. The cumulative incidence of successful VA-ECMO weaning between groups was analyzed using the Gray test. Subdistribution hazard ratios (sHR) and their corresponding 95% confidence intervals (CI) were calculated for the three outcomes using Fine and Gray competing risks regression.

Kaplan–Meier survival curves were constructed for patients in the levosimendan-ECMO and no-levosimendan-ECMO groups, with a focus on 6-month bridge-free survival. These curves were compared using the Wald test of a Cox proportional hazard model. Statistical significance was defined as a p-value < 0.05. All statistical analyses were conducted using R software version 4.4.1 (http://www.R-project.org).

## Results

### Patients

Over the study period, 320 patients treated with VA-ECMO for refractory cardiogenic shock were included, of whom 68 received levosimendan during their VA-ECMO course (Table 1). The baseline characteristics of the no-levosimendan group (n = 252) and the levosimendan group (n = 68) are detailed in Table 1. Propensity score matching yielded 47 unique pairs of patients with comparable characteristics (Table 1). In summary, before matching, patients in the levosimendan group were older (52 [43–60] vs. 57 [50–63] years),

**Table 1:** Baseline characteristics of the entire study cohort and after propensity score matching analysis

	Before matching			After matching		
	No levosimendan group (n = 252)	Levosimendan group (n = 68)	*P* value	No levosimendan group (n = 47)	Levosimendan group (n = 47)	Standardized mean difference
Age, *years* *	52 (43–60)	57 (50–63)	< 0.01	54 (45–62)	57 (48–63)	0.04
Male sex	180 (71)	50 (74)	0.73	36 (77)	35 (75)	0.04
BMI, *kg/m*^*2*^	25 (23–28)	26 (22–28)	0.94	26 (24–30)	25 (22–28)	0.32
SOFA score at ICU admission *	12 (9–16)	11 (7–13)	< 0.01	10 (7–14)	11 (8–13)	0.09
SAPS II score	71 (54–85)	61 (43–73)	< 0.01	59 (41–82)	62 (48–73)	0.06
Diabetes mellitus	23 (17)	10 (14)	0.65	6 (18)	7 (15)	0.09
Dyslipidemia	38 (28)	12 (18)	0.09	11 (33)	8 (17)	0.42
Peripheral artery disease	9 (7)	6 (9)	0.62	1 (3)	3 (6)	0.11
Cardiogenic shock origin *			< 0.01			
AMI	194 (77)	43 (63)		39 (83)	38 (81)	0.04
Myocarditis	30 (12)	3 (4)		2 (4)	3 (6)	0.09
Miscellaneous^&^	28 (11)	22 (32)		6 (13)	6 (13)	0.00
ECMO-CPR *	53 (21)	5 (7)	< 0.01	5 (6)	5 (6)	0.07
Pre-ECMO						
pH	7.25 (7.13–7.35)	7.28 (7.18–7.40)	0.09	7.31 (7.28–7.36)	7.30 (7.21–7.41)	0.39
Lactate, *mmol/L* *	7.1 (4.3–11.2)	4.7 (2.4–6.9)	< 0.01	5.0 (2.1–7.1)	4.9 (2.4–7.4)	0.09
Inotropic Score, *γ//kg/min*	104 (53–262)	25 (10–70)	< 0.01	66 (30–126)	26 (10–74)	0.31
AoVTI, *cm*	5 (2–7)	7 (5–10)	< 0.01	5 (4–7)	8 (5–10)	0.69
LVEF, *%*	10 (5–15)	15 (10–20)	< 0.01	10 (10–15)	15 (10–20)	0.39
Creatinine, *µmol/L*	148 (100–237)	125 (101–172)	0.15	171 (103–299)	123 (99–177)	0.36

AMI: acute myocardial infarction, Ao VTI: aortic velocity time integral, BMI: body mass index, CPR: cardiopulmonary resuscitation, ECMO: extracorporeal membrane oxygenation, ICU: intensive care unit, LVEF: left ventricular ejection fraction, SAPS II: Simplified Acute Physiology Score, SOFA: Sepsis-related Organ Failure Assessment

^*^Covariates selected in the propensity score

^&^ **Control Group:** Valvular cardiomyopathy (n = 2), non-ischemic storm rhythm (n = 3), toxic cardiomyopathy (n = 3), septic cardiomyopathy (n = 8), anaphylactic shock (n = 2), Takotsubo cardiomyopathy (n = 2), acute heart graft rejection (n = 4), unknown etiology (n = 4). **Levosimendan Group:** Valvular cardiomyopathy (n = 2), non-ischemic storm rhythm (n = 1), toxic cardiomyopathy (n = 4), septic cardiomyopathy (n = 6), Takotsubo cardiomyopathy (n = 2), acute heart graft rejection (n = 5), unknown etiology (n = 2)

while those in the no-levosimendan group had significantly higher Simplified Acute Physiology Score (SAPS II) and Sequential Organ Failure Assessment (SOFA) scores at ICU admission. These characteristics were well-balanced after matching. The etiologies of cardiogenic shock in the overall cohort included acute myocardial infarction (74%), myocarditis (10%), and other causes (16%). Both LVEF and AoVTI before VA-ECMO initiation were significantly lower in the no-levosimendan group compared to the levosimendan group (p < 0.05). Conversely, the inotropic score was significantly higher in the no-levosimendan group (104 [53–262] vs. 25 [10–70] µg/kg/min, p < 0.001). These differences persisted between the two groups even after matching (standardized mean difference ≥ 0.2) (Table 1).

### Levosimendan use according to VA-ECMO weaning status

**Fig. 1:**
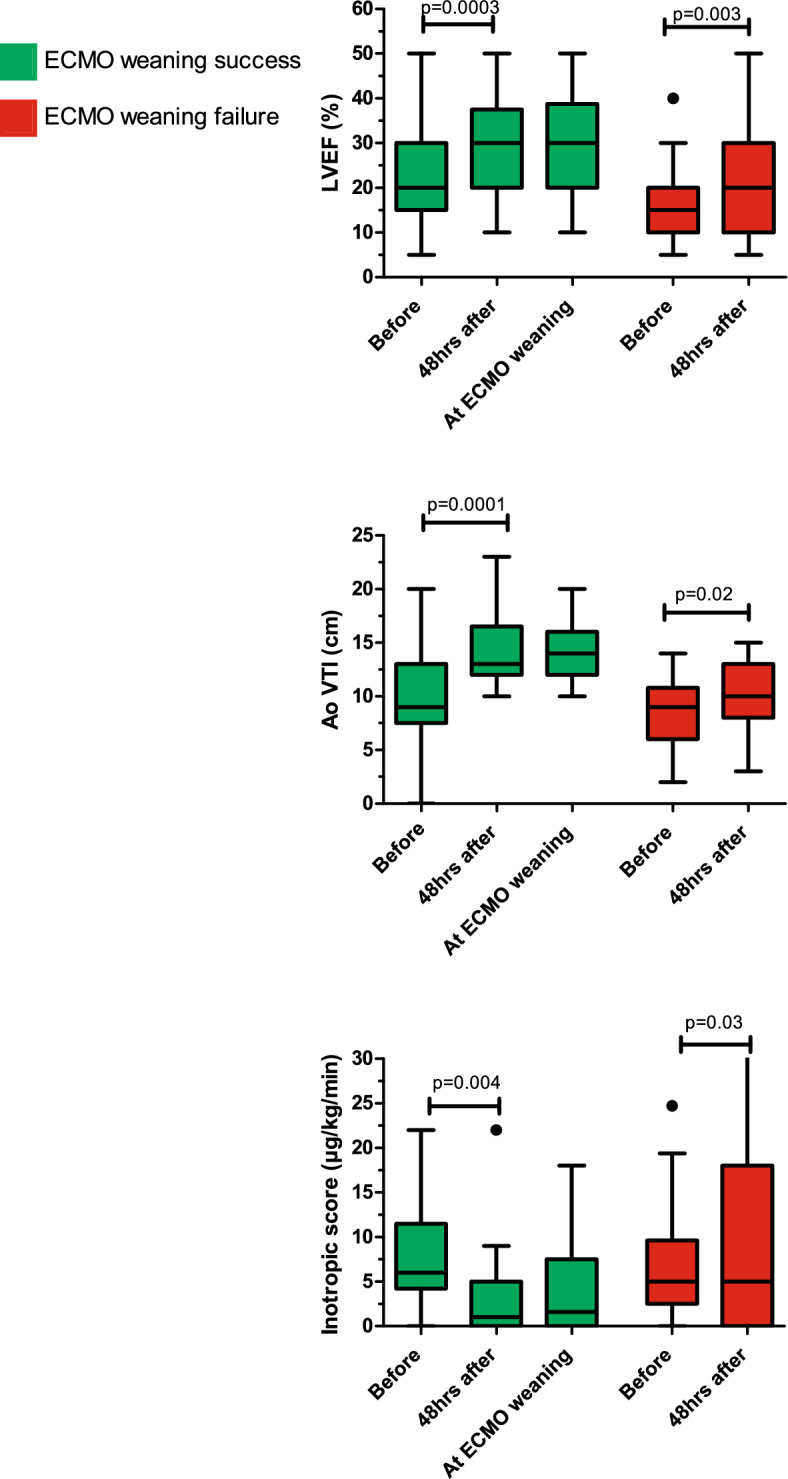
Evolution of the AoVTI, LVEF, and inotropic score before and 48 h after levosimendan on ECMO according to their ECMO-weaning status. *AO VTI: aortic velocity time integral, LVEF: left ventricular ejection fraction, VA-ECMO: venoarterial extracorporeal membrane oxygenation*

The median time to initiate levosimendan was 4 (3–9) days post-cannulation. Among the 68 patients who received levosimendan, 29 (43%) achieved successful VA-ECMO weaning 3 (1–6) days after initiation. In this group, LVEF improved significantly from 20% (15–30%) at the start of levosimendan infusion to 30% (20–37%) 48 h later (p < 0.001), accompanied by a significant increase in AoVTI during the same period (p < 0.001) (Fig. 1). Notably, LVEF and AoVTI also showed significant improvement in the weaning failure group, although this was not associated with a reduction in the inotropic score. Patients in the levosimendan group who failed VA-ECMO weaning were more frequently immunocompromised and had significantly lower LVEF at VA-ECMO initiation and before levosimendan infusion (Additional file 1).

### Tolerance of levosimendan

Adverse effects of levosimendan included supraventricular arrhythmias (13%) and severe hypotension (6%), with no significant difference observed between patients who experienced VA-ECMO weaning failure and those who achieved successful weaning (Table 2). Levosimendan infusion was prematurely discontinued in only one patient.

**Table 2:** VA-ECMO-related outcomes in patients who received levosimendan on ECMO according to their ECMO-successful weaning status

	VA-ECMO weaning success **(n = 29)**	VA-ECMO weaning failure (n = 39)	*P* value
VA-ECMO duration	8 (5–14)	14 (9–28)	< 0.01
Delay from ECMO to levosimendan infusion	4 (2–8)	5 (3–9)	0.35
Total artificial heart	0 (0)	0 (0)	1.00
LVAD	0 (0)	9 (23)	< 0.01
Heart transplant	0 (0)	3 (8)	0.25
Levosimendan-related complications			0.71
Supraventricular rhythm disorders	5 (17)	4 (10)	
Severe hypotension	1 (3)	3 (8)	
Premature stop of the levosimendan	0 (0)	1 (3)	
ICU-LOS	22 (14–32)	21 (12–36)	0.95

ICU: intensive care unit, LOS: length of stay, LVAD: left ventricular assist device, VA-ECMO: venoarterial-extracorporeal membrane oxygenation

### Levosimendan and outcomes

**Fig. 2:**
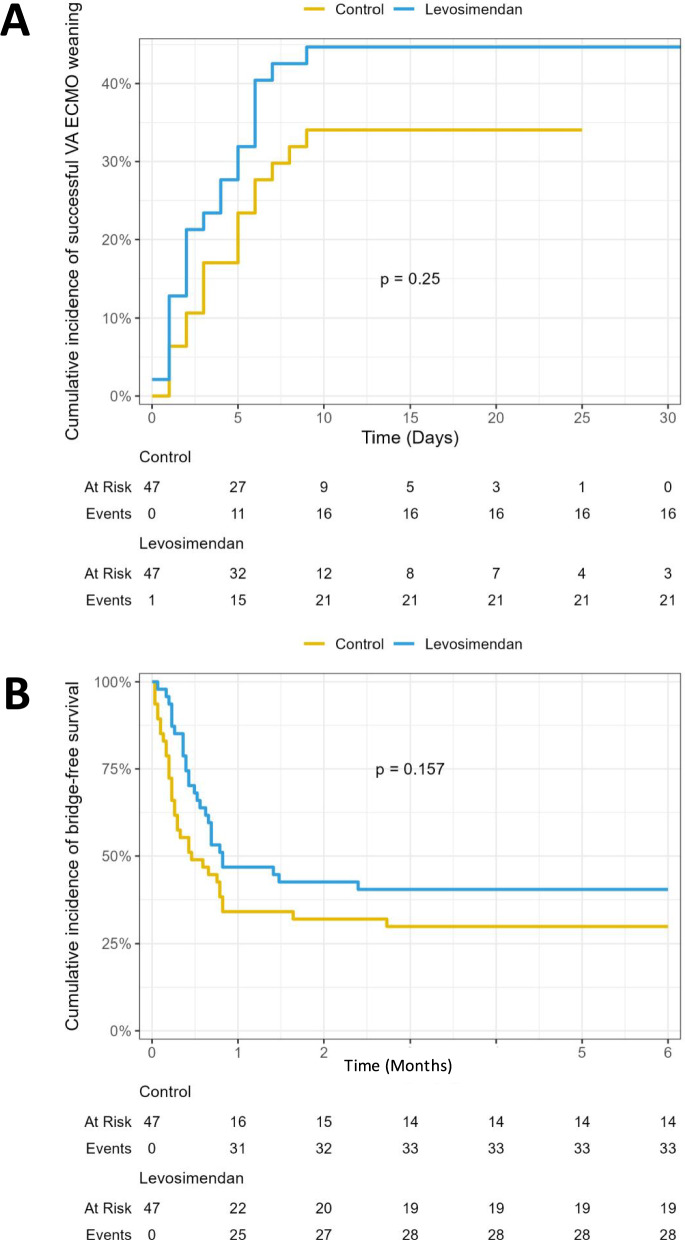
A) Cumulative incidence of successful VA-ECMO weaning, and B) cumulative incidence of survival free from a heart transplant, LVAD, or total artificial heart (i.e., bridge-free survival) at 6 months in the matched populations receiving and not receiving levosimendan on VA-ECMO

After matching, successful VA-ECMO weaning was achieved in 16 out of 47 patients (34%) in the no-levosimendan group and 21 out of 47 patients (45%) in the levosimendan group (sHR, 1.44 [95% CI, 0.77–2.70]; P = 0.25) (Fig. 2). Similarly, there were no significant differences between the groups in terms of bridge-to-heart transplant, LVAD, or total artificial heart (sHR 0.59 [95% CI, 0.28–1.26]; P = 0.18) or death (sHR 0.93 [95% CI, 0.46–1.87]; P = 0.85), which were the other components of successful VA-ECMO weaning. Among patients who did not achieve successful weaning, 27 were bridged from VA-ECMO to LVAD, heart transplant, or total artificial heart (16 in the no-levosimendan group and 11 in the levosimendan group) (Table 3). Notably, 15 patients (32%) in each group died on VA-ECMO or within 30 days after VA-ECMO weaning. Additionally, VA-ECMO duration, ICU length of stay, or 6-month survival were not significantly different between the two groups (Table 3).

**Table 3:** VA-ECMO-related outcomes in the matched population

	No levosimendan group (n = 47)	Levosimendan group (n = 47)	*P* value
VA-ECMO duration	10 (7–14)	11 (7–16)	0.15
ECMO-successful weaning	16 (34)	21 (45)	0.31
Causes of ECMO weaning failure			
Bridge within than 30 days from ECMO removal	16 (34)	11 (23)	0.25
LVAD	10 (21)	8 (17)	0.61
Heart transplant	3 (6)	3 (6)	1.00
Total artificial heart	3 (6)	0 (0)	0.07
Death on ECMO or within 30 days from ECMO removal	15 (32)	15 (32)	1.00
ICU-LOS	18 (12–43)	21 (13–34)	0.64
30-Day bridge free survival*	16 (34)	22 (47)	0.23
30-Day overall survival	30 (64)	29 (62)	0.84
6-month bridge free survival*	14 (30)	19 (40)	0.33
6-month overall survival	28 (60)	25 (53)	0.60

ICU: intensive care unit, LOS: length of stay, LVAD: left ventricular assist device, VA-ECMO: venoarterial-extracorporeal membrane oxygenation

*Bridge-free survival is defined as survival without a heart transplant, LVAD, or total artificial heart

## Discussion

In this bicentric cohort study involving non-postoperative patients undergoing VA-ECMO therapy, a 24-h continuous infusion of levosimendan did not lead to a statistically significant reduction in the time to successful VA-ECMO weaning. Furthermore, levosimendan did not demonstrate any benefits in decreasing VA-ECMO duration or improving survival rates at 30 days and 6 months post-ECMO.

Patients on ECMO are often subject to a significant inflammatory response and impaired endothelial function [1]. Levosimendan has the potential to markedly enhance endothelial function and boost the cardiac index in VA-ECMO patients with severely depressed left ventricular ejection fraction (LVEF < 25%) [26]. This beneficial effect on endothelial dysfunction may contribute to the hypothesized advantages of levosimendan in patients receiving VA-ECMO. Additionally, the inotropic properties of levosimendan, which operate through a different mechanism than dobutamine [4], may offer an appealing alternative for patients who are chronically exposed to dobutamine and, therefore at risk of down-regulation of cardiac beta-receptors [27]. Moreover, VA-ECMO may facilitate better hemodynamic tolerance of levosimendan. Reducing the duration of VA-ECMO could lead to limiting the risks of ECMO-related complications, such as cannula infections, bacteremia, and pulmonary edema, that impact patient outcomes [1]. For these reasons, levosimendan is frequently administered during VA-ECMO to aid in the weaning process potentially. According to data from a large registry of patients treated with levosimendan in France over a year, 13.6% of these patients received the drug in the context of VA-ECMO weaning [16].

However, the use of levosimendan in this clinical setting remains controversial. Our findings challenge those reported in previous studies involving non-operative VA-ECMO patients, which identified a strong correlation between levosimendan administration and successful VA-ECMO weaning, as well as improved short- and long-term survival outcomes [5, 9, 26]. Notably, Vally et al*.* demonstrated in a cohort of 38 propensity-matched patients that levosimendan exposure was the sole factor significantly associated with reduced VA-ECMO weaning failure rates [5]. However, more recent evidence from Guilherme et al*.* found no improvement in VA-ECMO weaning success rates in patients with refractory cardiogenic shock, based on a study comparing 48 patients receiving levosimendan to 78 control patients in an observational, single-center cohort [8]. In the context of VA-ECMO following cardiovascular surgery, Distelmaier et al*.* reported a significant association between levosimendan treatment and successful VA-ECMO weaning, as well as reductions in 30-day and long-term mortality in 179 patients who received levosimendan within the first 24 h of VA-ECMO initiation [6]. These findings were not confirmed in a recent emulated target trial involving 239 patients. Doubts were raised regarding the efficacy of levosimendan, as no significant association with VA-ECMO weaning success or mortality was observed in patients with refractory postcardiotomy cardiogenic shock [28]. These findings were consistent with results from randomized controlled trials among patients undergoing cardiac surgery without VA-ECMO [13–15].

Several meta-analyses, incorporating both operative and non-operative VA-ECMO patients, have shown higher success rates for VA-ECMO weaning in the levosimendan group [9, 11, 12]. The discrepancies between our findings and those of observational studies favoring levosimendan could be attributed to differences in methodology. Firstly, we applied a rigorous definition of "successful ECMO weaning," which required survival for 30 days post-weaning, as opposed to the 24 to 48-h survival period commonly used in previous studies [5, 6, 8, 28]. This shorter timeframe might have introduced a bias toward the levosimendan group. Secondly, our study was limited to patients with the potential for myocardial recovery, excluding those with dilated or end-stage cardiomyopathy, thereby yielding a more homogeneous cohort of non-operative VA-ECMO patients. Lastly, our matching process was conducted without replacement, ensuring that paired patients spent an equal amount of time on VA-ECMO. This critical methodological detail was not accounted for in previous studies [5, 8], potentially introducing an "immortality bias" in favor of patients on levosimendan.

Despite its good tolerability, the rate of premature discontinuation of levosimendan in our study was very low compared to recent randomized clinical trials [14], even though the incidence of adverse effects was similar. In this context, VA-ECMO support may facilitate the safe administration of higher doses of levosimendan (0.1 to 0.2 µg/kg/min) than those currently used, such as in the CHEETAH trial (e.g., 0.066 µg/kg/min) [14]. However, the effectiveness of a loading dose, which was not applied in our cohort of severe patients, remains uncertain.

The strengths of our study include its large bi-center cohort and longitudinal design, featuring a 6-month follow-up after ICU admission. However, there are also several limitations to consider. First, as a retrospective study, our findings are subject to the inherent biases of this study design, as residual confounders may not have been fully accounted for in our analyses. Additionally, the 11% higher rate of successful VA-ECMO weaning we observed in the levosimendan group did not reach statistical significance in our study which included 47 matched pairs of patients. This relatively small sample size may have limited the power to detect the efficacy of levosimendan in VA-ECMO weaning, which could become more evident with a larger patient cohort, as suggested by results of recent meta-analyses [9–11]. Second, residual baseline differences in disease severity between the two groups, even after matching, represent a potential limitation. Recognizing the possibility of residual confounding inherent to the observational design, we employed propensity score matching based on factors frequently associated with mortality. To further mitigate these limitations, this study is the first to account for immortal time bias and competing risks, providing a more precise estimate of the impact of levosimendan on weaning success. Finally, the timing of levosimendan administration was left to the treating physician's discretion, which may have introduced patient selection bias. Ensuring a similar duration of VA-ECMO in all matched pairs could have helped to mitigate this potential bias.

## Conclusion

In patients with non-postoperative cardiogenic shock supported by peripheral VA-ECMO, levosimendan was not associated with increased rates of successful VA-ECMO weaning or improved 30-day and 6-month bridge-free survival. These findings challenge current clinical practices and highlight the ongoing controversy surrounding this issue. To clarify the effectiveness and optimal timing of levosimendan in this specific population, results from double-blinded randomized controlled trials, such as LEVOECMO (NCT04728932) and Weanlevo (NCT04158674), are urgently needed.

## Supplementary Information

Additional file 1.: Baseline characteristics of the patients who received levosimendan on ECMO according to their ECMO-successful weaning status.

## Data Availability

The datasets analyzed during the current study are available from the corresponding author upon reasonable request.

## Abbreviations

AoVTI: Aortic velocity time integral
ICU: Intensive care unit
LVAD: Left ventricular assist device
LVEF: Left ventricular ejection fraction
SAPS II: Simplified Acute Physiology Score II
sHR: Subdistribution hazard ratios
SOFA: Sequential Organ Failure Assessment
VA-ECMO: Venoarterial extracorporeal membrane oxygenation

## References

[1] Abrams D, Combes A, Brodie D. Extracorporeal membrane oxygenation in cardiopulmonary disease in adults. J Am Coll Cardiol. 2014;63:2769–78. 24814488 10.1016/j.jacc.2014.03.046

[2] Ostadal P, Rokyta R, Karasek J, Kruger A, Vondrakova D, Janotka M, et al. Extracorporeal Membrane Oxygenation in the Therapy of Cardiogenic Shock: Results of the ECMO-CS Randomized Clinical Trial. Circulation. 2023;147:454–64. 36335478 10.1161/CIRCULATIONAHA.122.062949

[3] Thiele H, Zeymer U, Akin I, Behnes M, Rassaf T, Mahabadi AA, et al. Extracorporeal Life Support in Infarct-Related Cardiogenic Shock. N Engl J Med. 2023;389:1286–97. 37634145 10.1056/NEJMoa2307227

[4] Pathak A, Lebrin M, Vaccaro A, Senard JM, Despas F. Pharmacology of levosimendan: inotropic, vasodilatory and cardioprotective effects. J Clin Pharm Ther. 2013;38:341–9. 23594161 10.1111/jcpt.12067

[5] Vally S, Ferdynus C, Persichini R, Bouchet B, Braunberger E, Lo Pinto H, et al. Impact of levosimendan on weaning from peripheral venoarterial extracorporeal membrane oxygenation in intensive care unit. Ann Intensive Care. 2019;9:24. 30707314 10.1186/s13613-019-0503-1 PMC6358626

[6] Distelmaier K, Roth C, Schrutka L, Binder C, Steinlechner B, Heinz G, et al. Beneficial effects of levosimendan on survival in patients undergoing extracorporeal membrane oxygenation after cardiovascular surgery. Br J Anaesth. 2016;117:52–8. 27317704 10.1093/bja/aew151 PMC4913403

[7] Affronti A, di Bella I, Carino D, Ragni T. Levosimendan may improve weaning outcomes in venoarterial ECMO patients. ASAIO J Am Soc Artif Intern Organs. 1992;2013(59):554–7. 10.1097/MAT.0b013e3182a4b32e 24172260

[8] Guilherme E, Jacquet-Lagrèze M, Pozzi M, Achana F, Armoiry X, Fellahi J-L. Can levosimendan reduce ECMO weaning failure in cardiogenic shock?: a cohort study with propensity score analysis. Crit Care Lond Engl. 2020;24:442. 10.1186/s13054-020-03122-y PMC7367381 32677985

[9] Liu Y, Zhang L, Yao Y, Li Y, Qin W, Li Y, et al. Effects of levosimendan on the outcome of veno-arterial extracorporeal membrane oxygenation: a systematic review and meta-analysis. Clin Res Cardiol Off J Ger Card Soc. 2024;113:509–21. 10.1007/s00392-023-02208-1 37217802

[10] Ng KT, Chan XL, Tan W, Wang CY. Levosimendan use in patients with preoperative low ejection fraction undergoing cardiac surgery: A systematic review with meta-analysis and trial sequential analysis. J Clin Anesth. 2019;52:37–47. 30172838 10.1016/j.jclinane.2018.08.019

[11] Yang B, Zhao T, Guo B, Li Y. Short-term effects of levosimendan use for venoarterial extracorporeal membrane oxygenation: A systematic review and meta-analysis. Perfusion. 2023;38:305–12. 34689640 10.1177/02676591211051860

[12] Bertini P, Paternoster G, Landoni G, Falcone M, Nocci M, Costanzo D, et al. Beneficial effects of levosimendan to wean patients from VA-ECMO: a systematic review and meta-analysis. Minerva Cardiol Angiol. 2023;71:564–74. 35687316 10.23736/S2724-5683.22.06054-9

[13] Cholley B, Caruba T, Grosjean S, Amour J, Ouattara A, Villacorta J, et al. Effect of Levosimendan on Low Cardiac Output Syndrome in Patients With Low Ejection Fraction Undergoing Coronary Artery Bypass Grafting With Cardiopulmonary Bypass: The LICORN Randomized Clinical Trial. JAMA. 2017;318:548–56. 28787507 10.1001/jama.2017.9973 PMC5817482

[14] Landoni G, Lomivorotov VV, Alvaro G, Lobreglio R, Pisano A, Guarracino F, et al. Levosimendan for Hemodynamic Support after Cardiac Surgery. N Engl J Med. 2017;376:2021–31. 28320259 10.1056/NEJMoa1616325

[15] Mehta RH, Leimberger JD, van Diepen S, Meza J, Wang A, Jankowich R, et al. Levosimendan in Patients with Left Ventricular Dysfunction Undergoing Cardiac Surgery. N Engl J Med. 2017;376:2032–42. 28316276 10.1056/NEJMoa1616218

[16] Cholley B, Bojan M, Guillon B, Besnier E, Mattei M, Levy B, et al. Overview of the current use of levosimendan in France: a prospective observational cohort study. Ann Intensive Care. 2023;13:69. 37552372 10.1186/s13613-023-01164-3 PMC10409690

[17] Muller G, Flecher E, Lebreton G, Luyt C-E, Trouillet J-L, Bréchot N, et al. The ENCOURAGE mortality risk score and analysis of long-term outcomes after VA-ECMO for acute myocardial infarction with cardiogenic shock. Intensive Care Med. 2016;42:370–8. 26825953 10.1007/s00134-016-4223-9

[18] Bréchot N, Demondion P, Santi F, Lebreton G, Pham T, Dalakidis A, et al. Intra-aortic balloon pump protects against hydrostatic pulmonary oedema during peripheral venoarterial-extracorporeal membrane oxygenation. Eur Heart J Acute Cardiovasc Care. 2018;7:62–9. 28574276 10.1177/2048872617711169

[19] Dangers L, Bréchot N, Schmidt M, Lebreton G, Hékimian G, Nieszkowska A, et al. Extracorporeal Membrane Oxygenation for Acute Decompensated Heart Failure. Crit Care Med. 2017;45:1359–66. 28471885 10.1097/CCM.0000000000002485

[20] Ponikowski P, Voors AA, Anker SD, Bueno H, Cleland JGF, Coats AJS, et al. 2016 ESC Guidelines for the diagnosis and treatment of acute and chronic heart failure: The Task Force for the diagnosis and treatment of acute and chronic heart failure of the European Society of Cardiology (ESC)Developed with the special contribution of the Heart Failure Association (HFA) of the ESC. Eur Heart J. 2016;37:2129–200. 27206819 10.1093/eurheartj/ehw128

[21] Møller JE, Sionis A, Aissaoui N, Ariza A, Bělohlávek J, De Backer D, et al. Step by step daily management of short-term mechanical circulatory support for cardiogenic shock in adults in the intensive cardiac care unit: a clinical consensus statement of the Association for Acute CardioVascular Care of the European Society of Cardiology SC, the European Society of Intensive Care Medicine, the European branch of the Extracorporeal Life Support Organization, and the European Association for Cardio-Thoracic Surgery. Eur Heart J Acute Cardiovasc Care. 2023;12:475–85. 37315190 10.1093/ehjacc/zuad064

[22] Bréchot N, Luyt C-E, Schmidt M, Leprince P, Trouillet J-L, Léger P, et al. Venoarterial Extracorporeal Membrane Oxygenation Support for Refractory Cardiovascular Dysfunction During Severe Bacterial Septic Shock. Crit Care Med. 2013; 10.1097/CCM.0b013e31828a2370 23563585

[23] Aissaoui N, El-Banayosy A, Combes A. How to wean a patient from veno-arterial extracorporeal membrane oxygenation. Intensive Care Med. 2015;41:902–5. 25619488 10.1007/s00134-015-3663-y

[24] Aissaoui N, Luyt C-E, Leprince P, Trouillet J-L, Léger P, Pavie A, et al. Predictors of successful extracorporeal membrane oxygenation (ECMO) weaning after assistance for refractory cardiogenic shock. Intensive Care Med. 2011;37:1738–45. 21965097 10.1007/s00134-011-2358-2

[25] Austin PC. Some methods of propensity-score matching had superior performance to others: results of an empirical investigation and Monte Carlo simulations. Biom J Biom Z. 2009;51:171–84. 10.1002/bimj.200810488 19197955

[26] Sangalli F, Avalli L, Laratta M, Formica F, Maggioni E, Caruso R, et al. Effects of Levosimendan on Endothelial Function and Hemodynamics During Weaning From Veno-Arterial Extracorporeal Life Support. J Cardiothorac Vasc Anesth. 2016;30:1449–53. 27495966 10.1053/j.jvca.2016.03.139

[27] Vallet B, Dupuis B, Chopin C. Dobutamine: mechanisms of action and use in acute cardiovascular pathology. Ann Cardiol Angeiol (Paris). 1991;40:397–402. 1859148

[28] Massol J, Simon-Tillaux N, Tohme J, Hariri G, Dureau P, Duceau B, et al. Levosimendan in patients undergoing extracorporeal membrane oxygenation after cardiac surgery: an emulated target trial using observational data. Crit Care Lond Engl. 2023;27:51. 10.1186/s13054-023-04328-6 PMC9906922 36750852

