# Supplementary material for: Does Levosimendan hasten veno-arterial ECMO weaning? A propensity score matching analysis
Source: Ann Intensive Care. 2025 Apr 3;15:48. doi: 10.1186/s13613-025-01457-9 (PMC11968594; doi:10.1186/s13613-025-01457-9)
Supplement: Supplementary file 1 — Additional file 1.: Baseline characteristics of the patients who received levosimendan on ECMO according to their ECMO-successful weaning status. [file 13613_2025_1457_MOESM1_ESM.docx]

**Additional file 1: Baseline characteristics of the patients who received levosimendan on ECMO according to their ECMO-successful weaning status.**

|  | **VA-ECMO weaning success**  **(n=29)** | **VA-ECMO weaning failure (n=39)** | ***P* value** |
| --- | --- | --- | --- |
| Age, *years* | 60 (50-66) | 57 (50-61) | 0.25 |
| Male sex | 21 (72) | 29 (74) | 0.86 |
| BMI, *kg/m^2^* | 27 (23-29) | 25 (22-28) | 0.20 |
| Immunosuppression | 2 (7) | 11 (28) | 0.03 |
| Diabetes mellitus | 5 (17) | 5 (13) | 0.73 |
| Dyslipidemia | 3 (10) | 9 (23) | 0.17 |
| Peripheral artery disease | 0 (0) | 6 (15) | 0.03 |
| SOFA score at ICU admission | 10 (7-13) | 11 (8-13) | 0.28 |
| SAPS II score | 62 (45-76) | 60 (42-70) | 0.37 |
| **Cardiogenic shock origin** |  |  | 0.51 |
| AMI | 16 (55) | 27 (69) |  |
| Myocarditis | 2 (7) | 1 (3) |  |
| Other | 11 (38) | 11 (28) |  |
| VA-ECMO-CPR | 1 (3) | 4 (10) | 0.38 |
| **At ECMO Start** |  |  |  |
| pH | 7.32 (7.25-7.44) | 7.25 (7.17-7.34) | 0.06 |
| Lactate, *mmol/L* | 4.6 (2.3-5.3) | 4.9 (3.1-7.4) | 0.28 |
| Inotropic Score, *γ/kg/min* | 25 (12-69) | 25 (10-72) | 0.93 |
| AoVTI, *cm* | 9 (6-10) | 7 (5-8) | 0.04 |
| LVEF, *%* | 20 (15-30) | 10 (10-15) | <0.01 |
| Creatinine, *µmol/L* | 119 (100-169) | 125 (103-180) | 0.61 |
| Billirubin, *mmol/L* | 12 (8-18) | 10 (5-16) | 0.14 |
| Renal replacement therapy | 1 (3) | 1 (3) | 1.00 |
| **At levosimendan infusion** |  |  |  |
| Delay VA-ECMO-levosimendan, *days* | 4 (2-8) | 5 (3-9) | 0.35 |
| Dobutamine | 21 (72) | 31 (82) | 0.37 |
| Dose, *γ/kg/min* | 3.3 (0-5) | 5 (2.5-6.1) | 0.25 |
| Norepinephrine | 9 (31) | 6 (16) | 0.14 |
| Dose, *γ/kg/min* | 0 (0-0.06) | 0 (0-0) | 0.12 |
| Inotropic Score, *γ/kg/min* | 5.0 (4.7-11.3) | 5.0 (2.7-9.2) | 0.33 |
| ECMO flow, *L/min* | 2.5 (2.2-3.1) | 2.6 (2.2-3.2) | 0.53 |
| AoVTI, *cm* | 9.0 (7.5-12.7) | 9.0 (6.4-10.2) | 0.22 |
| LVEF, *%* | 20 (15-30) | 15 (10-20) | 0.02 |
| pH | 7.51 (7.47-7.54) | 7.48 (7.42-7.52) | 0.08 |
| Lactate, *mmol/L* | 1.2 (0.8-1.4) | 1.3 (1.0-1.6) | 0.36 |
| Billirubin, *mmol/L* | 13 (8-20) | 12 (9-24) | 0.72 |
| Creatinine, *µmol/L* | 101 (60-131) | 119 (80-180) | 0.13 |

*AMI: acute myocardial infarction, AO VTI: aortic velocity time integral, BMI: body mass index, CPR: cardiopulmonary resuscitation, ICU: intensive care unit, LOS: length of stay, LVAD: left ventricular assist device, LVEF: left ventricular ejection fraction, SAPS II:* Simplified Acute Physiology*Score, SOFA: Sepsis-related Organ Failure Assessment: VA-ECMO: venoarterial-extracorporeal membrane oxygenation*
